# Histones Methyltransferase NSD3 Inhibits Lung Adenocarcinoma Glycolysis Through Interacting with PPP1CB to Decrease STAT3 Signaling Pathway

**DOI:** 10.1002/advs.202400381

**Published:** 2024-08-09

**Authors:** Yanling Zhou, Xintong Peng, Cheng Fang, Xin Peng, Jianing Tang, Zuli Wang, Yao Long, Jielin Chen, Yuanhao Peng, Zewen Zhang, Yanmin Zhou, Jun Tang, Jingzhong Liao, Desheng Xiao, Yongguang Tao, Ying Shi, Shuang Liu

**Affiliations:** ^1^ Department of Oncology Institute of Medical Sciences National Clinical Research Center for Geriatric Disorders Institue of Medical Sciences Xiangya Hospital, Central South University Changsha Hunan 410008 China; ^2^ Department of Hematology Xiangya Hospital, Central South University Changsha Hunan 410008 China; ^3^ Key Laboratory of Carcinogenesis and Cancer Invasion Ministry of Education, Department of Pathology Xiangya Hospital, Central South University Changsha Hunan 410008 China; ^4^ Cancer Research Institute School of Basic Medicine Central South University Changsha Hunan 410028 China; ^5^ Department of Cardiac Surgery Xiangya Hospital, Central South University Changsha Hunan 410008 China; ^6^ Department of Pathology Xiangya Hospital, Central South University Changsha Hunan 410008 China; ^7^ Department of Liver Surgery Xiangya Hospital, Central South University Changsha Hunan 410008 China; ^8^ Center for Tissue Engineering and Stem Cell Research Guizhou Medical University Guiyang Guizhou 561113 China; ^9^ Department of Laboratory Medicine Xiangya Hospital, Central South University Changsha Hunan 410008 China

**Keywords:** glycolysis, histones methyltransferase, lung adenocarcinoma, STAT3 signaling pathway

## Abstract

Histones methyltransferase NSD3 targeting H3K36 is frequently disordered and mutant in various cancers, while the function of NSD3 during cancer initiation and progression remains unclear. In this study, it is proved that downregulated level of NSD3 is linked to clinical features and poor survival in lung adenocarcinoma. In vivo, NSD3 inhibited the proliferation, immigration, and invasion ability of lung adenocarcinoma. Meanwhile, NSD3 suppressed glycolysis by inhibiting HK2 translation, transcription, glucose uptake, and lactate production in lung adenocarcinoma. Mechanistically, as an intermediary, NSD3 binds to PPP1CB and p‐STAT3 in protein levels, thus forming a trimer to dephosphorylate the level of p‐STAT3 by PPP1CB, leading to the suppression of HK2 transcription. Interestingly, the phosphorylation function of PPP1CB is related to the concentration of carbon dioxide and pH value in the culture environment. Together, this study revealed the critical non‐epigenetic role of NSD3 in the regulation of STAT3‐dependent glycolysis, providing a piece of compelling evidence for targeting the NSD3/PPP1CB/p‐STAT3 in lung adenocarcinoma.

## Graphical abstract

This research clarifies the crucial role of NSD3 in lung adenocarcinoma that is correlated with NSD3/PPP1CB/p‐STAT3 dimer formation, revealing the correlation between non‐epigenetic functions of NSD3 and tumor cell metabolism, suggesting the potential of NSD3 in targeted therapy in lung adenocarcinoma.

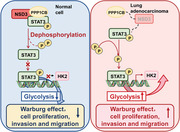

## 1 Introduction

Lung cancer remains a leading cause of cancer‐associated mortality in the United States and worldwide.^[^ ^1^ ^]^ Lung adenocarcinoma (LUAD), a common subtype of lung cancer, accounts for ≈40% of lung cancer.^[^ ^2^ ^]^ To meet the growth needs of tumor cells, changes in energy metabolism often occur, characterized by tumor cells preferring to obtain ATP through glycolysis even in aerobic conditions, also called the Warburg effect.^[^ ^3^ ^]^ The Warburg effect not only provides cancer cells with sufficient ATP synthesis and biosynthesis but also confers signaling functions in tumorigenesis.^[^ ^4^ ^]^ A growing number of research indicate that active epigenetic alterations have been linked to the process of Warburg effect.^[^ ^5^, ^6^ ^]^ Numerous findings have proved that histone methyltransferases (HMT) targeting H3K4 and H3K27 play a crucial role in the process of glycolysis and Warburg effect.^[^ ^7^, ^8^, ^9^, ^10^, ^11^, ^12^ ^]^ At the same time, recent genomic sequencing and numerous basic research implying that the alteration of HMTs might affect the tumor progression and patients’ prognosis of lung adenocarcinoma.^[^ ^13^, ^14^, ^15^ ^]^

Nuclear SET domain‐containing protein 3 (NSD3/WHSC1L1), which is located in 8p11.2, promotes methylation of H3K36.^[^ ^16^ ^]^ NSD3 is not only essential for the process of development in critical neural crest transcription factors but also vital to tumorigenesis.^[^ ^17^ ^]^ In detail, NSD3 can accelerate the expression of the Notch‐related signaling pathway through its methylase activity, thereby promoting the nuclear accumulation of NICD and the transcriptional repression of E‐cadherin, which facilitates the epithelial‐mesenchymal transition (EMT) and the occurrence and metastasis of breast cancer.^[^ ^18^ ^]^ Sequencing studies of lung squamous cell carcinoma (LUSC) proteogenomics identified NSD3 as an essential alternative driver molecule in patients with FGFR1 overexpression.^[^ ^19^ ^]^ Although studies have revealed that NSD3 plays a necessary epigenetic regulatory mechanism in most tumors, whether NSD3 is involved in the development and progression of lung adenocarcinoma remains unclear.

The warburge effect is achieved by the upregulation of glycolysis‐related enzymes and transporters in cancer. The conversion of glucose to glucose‐6‐phosphate (G6P) catalyzed by hexokinase 2 (HK2) is the first irreversible rate‐limiting reaction of glycolysis.^[^ ^20^ ^]^ A large number of studies have shown that HK2 expression is significantly up‐regulated in a variety of tumors, such as prostate cancer, breast cancer, lung cancer, kidney cancer, liver cancer, and colorectal cancer.^[^ ^21^, ^22^, ^23^ ^]^ Further mechanistic studies have shown that abnormally elevated HK2 expression mediates the Warburg effect in tumor cells, thereby promoting tumor cell proliferation, metastasis, and drug resistance.^[^ ^24^, ^25^, ^26^ ^]^ Mechanistically, the transcription of HK2 is affected by a variety of transcription factors and signaling pathways such as C‐MYC^[^ ^27^ ^]^, HIF‐1α,^[^ ^28^ ^]^ p53,^[^ ^26^ ^]^STAT3,^[^ ^29^ ^]^ et al,. However, the mechanisms underlying the transcription regulation of HK2 in lung adenocarcinoma need to be further elucidated.

In this study, we comprehensively and intimately clarified the function of NSD3 in lung adenocarcinoma, a type of cancer with low expression of NSD3. We found that NSD3 inhibited the proliferation, migration, invasion, and the Warburg effect in lung adenocarcinoma. NSD3 formed protein trimer with PPP1CB and p‐STAT3, thus stimulating p‐STAT3 dephosphorylation by PPP1CB to suppress the transcription of HK2, which ultimately inhibited the Warburg effect in lung adenocarcinoma. In summary, our studies explore the vital role of NSD3 non‐epigenetic regulatory effects in lung adenocarcinoma progression and metabolism, indicating that NSD3 as a promising therapeutic target for lung adenocarcinoma.

## 2 Results

### 2.1 NSD3 was Downregulated in LUAD and Correlated with Preferable Survival

**Figure 1:**
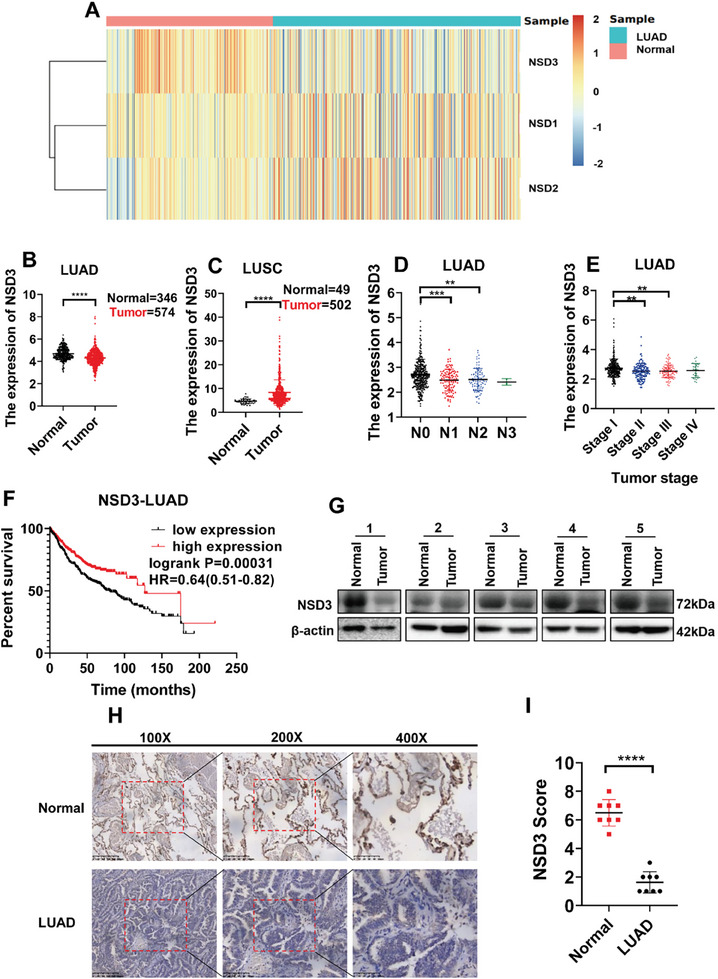
The validation and function prediction of NSD3 in LUAD patients from TCGA‐LUAD dataset. A) The heatmaps of Expression of NSD family in TCGA lung adenocarcinoma (with normal and tumor samples). B) Expression of NSD3 in TCGA lung adenocarcinoma (with normal and tumor samples). C) Expression of NSD3 in TCGA lung squamous cell carcinoma (with normal and tumor samples). D) Expression of NSD3 in TCGA lung adenocarcinoma (with tumor N stage). E) Expression of NSD3 in TCGA lung adenocarcinoma (with tumor stage). F) Kaplan–Meier curves for overall survival rates associated with samples measured here for lung adenocarcinoma. G) Western blot of NSD3 expression in lung adenocarcinoma paired with normal lung tissues. H) Immunohistochemistry of NSD3 expression in lung adenocarcinoma paired with normal lung tissues. I) Immunohistochemistry quantification for NSD3 level of lung adenocarcinoma paired with normal lung tissues (n = 8). ^*^ *p*  <0.05; ^**^ *p* < 0.01; ^***^ *p* < 0.001; and ^****^ *p* < 0.0001.

We first analyzed the expression of NSD family members in LUAD and normal tissues, which indicated that NSD3 was low expressed in LAUD, different from other members (**Figure** **1**A,B). The previous study showed that NSD3 is amplified in LUSC, and the overexpression of NSD3 promotes the tumorigenesis of LUSC (Figure 1C).^[^ ^30^ ^]^ Moreover, the amplification frequency of NSD3 in LUSC was significantly higher than in LUAD (Figure S1A,B, Supporting Information). At the same time, we analyzed the mutation rates of H3K36 enzymes (including NSD1, NSD2, NSD3, ASH1L, SMYD2, SETDB2, SETD3, and SETMAR) in lung adenocarcinoma, which showed that the amplification mutation frequency of H3K36 enzymes was at a low level (Figure S2, Supporting Information). Correspondingly, the expression of NSD3 negatively correlated with N and tumor stages (Figure 1D,E). Moreover, Kaplan‐Meier survival curves suggested that patients with high levels of NSD3 possessed lower survival rates (Figure 1F). To further validate the results from TCGA databases, we collected five pairs of LUAD clinical samples and para‐carcinoma tissues to test the protein levels of NSD3 by Western Blot (Figure 1G) and immunohistochemistry (IHC) (Figure 1H,I), which confirmed that NSD3 was downregulated in LUAD tissue than in peritumoral tissue. Together, the low amplification frequency and low copy numbers of NSD3 in LUAD were significantly correlated with a preferable survival.

### 2.2 NSD3 Inhibited Glycolysis Through HK2 in LUAD

**Figure 2:**
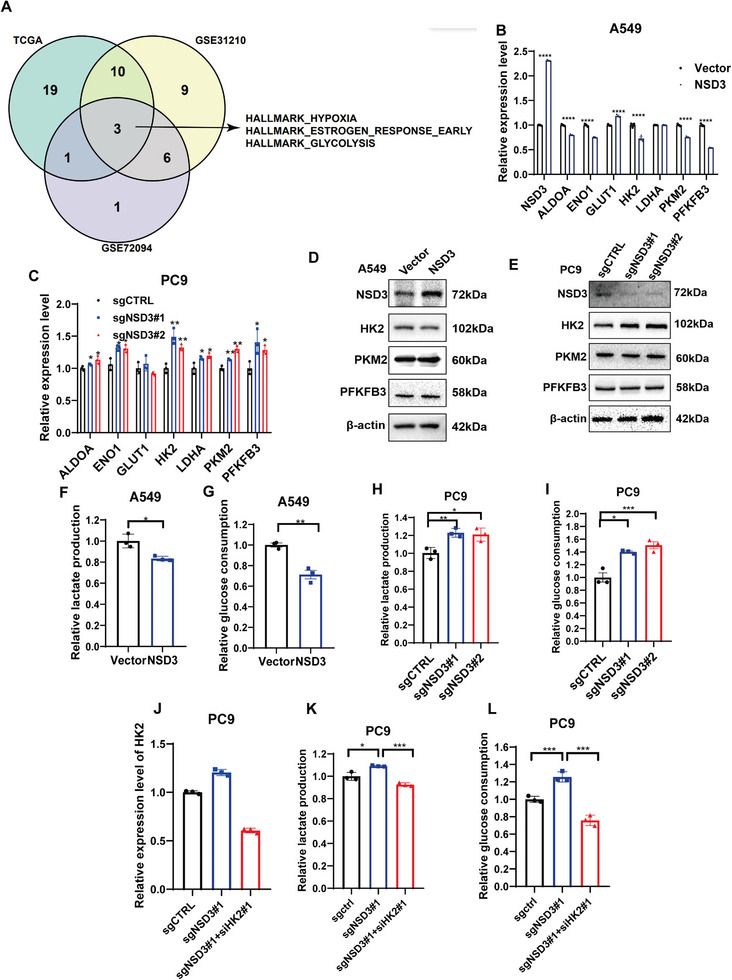
NSD3 regulates glycolysis in LUAD through HK2. A) Lung adenocarcinoma in each dataset (TCGA, GSE31210 andGSE72094) were significantly correlated with NSD3 expression (*p* < 0.05, *R* > 0.1) intersection of pathways:HALLMARK_HYPOXIA, HALLMARK_ESTROGEN_RESPONSE_EA RLY, HALLMARK_GLYCOLYSIS. B qRT‐PCR analysis of eight glycolysis enzymes in NSD3‐overexpressed A549 cells (n = 3 each). C) qRT‐PCR analysis of eight glycolysis enzymes in NSD3‐knockout PC9 cells (n = 3 each). D) Western blot analysis of NSD3 and HK2 in NSD3‐overexpressed A549 cells. E) Western blot analysis of NSD3 and HK2 in NSD3‐knockout PC9 cells. F) Relative lactate production in vector or NSD3‐overexpressed A549 cells (n = 3 each). G. Relative glucose consumption in vector or NSD3‐overexpressed A549 cells (n = 3 each). H) Relative lactate production in control or NSD3‐knockout PC9 cells (n = 3 each). I. Relative glucose consumption in control or NSD3‐knockout PC9 cells (n = 3 each). J) qRT‐PCR analysis of HK2 transcription in NSD3‐knockout PC9 cells interfered with HK2 (n = 3 each). K) Relative lactate production in control or NSD3‐knockout PC9 cells interfered with HK2 (n = 3 each). L) Relative glucose consumption in control or NSD3‐knockout PC9 cells interfered with HK2 (n = 3 each). ^*^ *p*  < 0.05; ^**^ *p* < 0.01; ^***^ *p* < 0.001; and ^****^ *p* < 0.0001.

To further determine the function of NSD3 in cancer, we used ssGSEA to analyze the correlation between biology process and NSD3 expression in lung adenocarcinoma based on TCGA lung adenocarcinoma database, GSE31210 and GSE72094 datasets, which proved that NSD3 are negative correlated with glycolysis (**Figure** **2**A; Figure S3A,B, Supporting Information). Therefore, we tested eight glycolysis enzymes mRNA levels when NSD3 was overexpressed or knockout, indicating that almost all glycolysis enzymes were inhibited by NSD3 (Figure 2B,C). Previous findings have shown that HK2 (hexokinase 2), PFKFB3 (6‐phosphofructo‐2‐kinase/fructose‐2,6‐biphosphatase 3), and PKM2 (Pyruvate kinase M2) are both key glycolysis rate‐limiting enzymes.^[^ ^31^ ^]^ Hence, we detected their protein expressions changes by Western blot, and we found that HK2 is upregulated in NSD3‐knockout PC9 cells and downregulated in NSD3‐overexpressed A549 cells (Figure 2D,E). In contrast, the expressions of PKM2 and PFKFB3 were not affected by NSD3 levels (Figure 2D,E). Next, we detected lactate production and glucose consumption to identify the function of NSD3 on the LUAD cell Warburg effect of LUAD cells. The results showed that overexpression of NSD3 inhibited the levels of lactate production and glucose consumption in the A549 LUAD cell (Figure 2F,G). Meanwhile, NSD3 knockout promoted the lactate production and glucose consumption of the PC9 LUAD cells (Figure 2H,I). To detect whether NSD3‐influenced glycolysis levels in lung adenocarcinoma cells are dependent on HK2 expression, the siRNA were used to interfere with HK2 transcription and translation in NSD3 knockout PC9 LUAD cells (Figure 2J). The results indicates that increased lactate production and glucose consumption in NSD3 knockout PC9 LUAD cells were reversed by silencing HK2 (Figure 2K,L). Overall, the findings indicated that NSD3 inhibits glycolysis in LUAD by inhibiting HK2.

### 2.3 NSD3 Suppressed HK2 via the Down‐Regulation of STAT3 Phosphorylation

**Figure 3:**
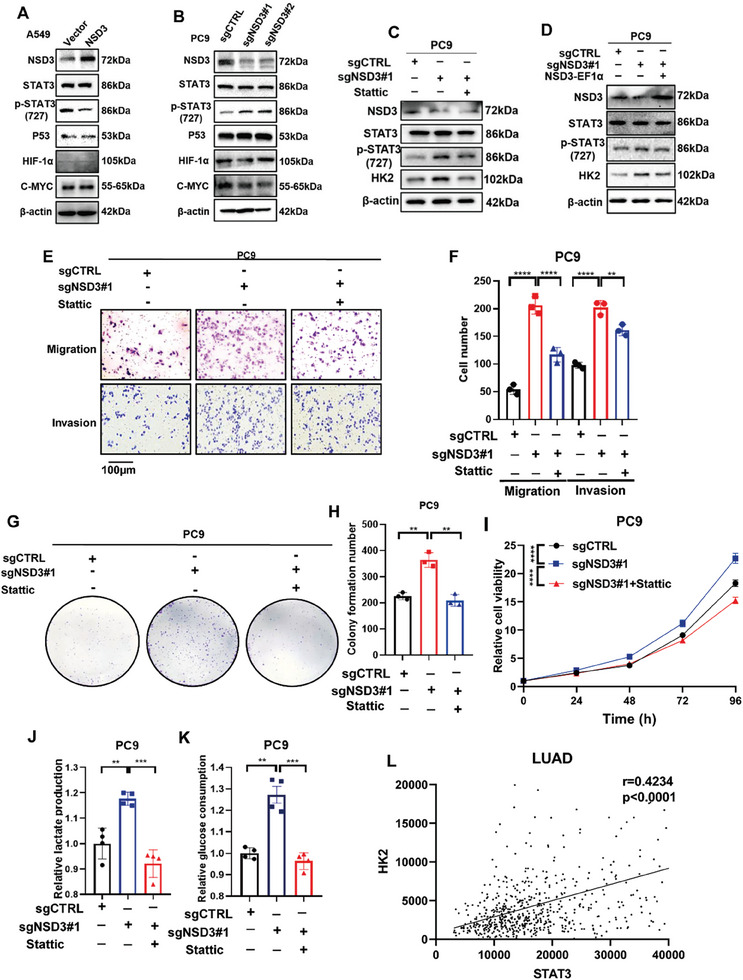
NSD3 regulates the levels of HK2 via STAT3 activation. A) Western blot analysis was performed to detect the four transcription regulation factors of HK2 in vector or NSD3‐overexpressed A549 cells. B) Western blot analysis was performed to detect the four transcription regulation factors of HK2 in control or NSD3‐knockout PC9 cells. C) After treatment with Stattic (5 µM) for 24 h, western blot analysis was performed to detect the expression of NSD3, p‐STAT3, and HK2 in control or NSD3‐knockout PC9 cells. D) After transfected with NSD3 plasmid, western blot analysis was performed to detect the expression of NSD3, p‐STAT3, and HK2 in control or NSD3‐knockout PC9 cells. E) After treatment with Stattic (5 µM) for 24 h, migration and invasion assay were performed to test the migration and invasion ability in control or NSD3‐knockout PC9 cells. F) Qualification of (E) (n = 3 each). G) After treatment with Stattic (5 µM) for 24 , colony formation assay was performed to test the proliferation ability in control or NSD3‐knockout PC9 cells. H) Qualification of (G) (n = 3 each). I. After treatment with Stattic (5 µM) for 24 h, CCK8 assay was performed to test the proliferation ability in control or NSD3‐knockout PC9 cells. Data are represented as mean ± S.E.M. (n = 5). P values are obtained by two‐way ANOVA. J) Relative lactate production in control or NSD3‐knockout PC9 cells with treatment of Stattic (5 µM) for 24 h (n = 4 each). K) Relative glucose consumption in control or NSD3‐knockout PC9 cells with treatment of Stattic (5 µM) for 24 h (n = 4 each). L. The correlation between HK2 and STAT3 in lung adenocarcinoma was analyzed. ^*^ *p* < 0.05; ^**^ *p* < 0.01; ^***^ *p* < 0.001; and ^****^ *p* < 0.0001.

Previous studies have proved that four molecular factors, including STAT3, p53, HIF‐1α, and C‐MYC, play a crucial role in HK2 transcription regulation in human cells.^[^ ^28^, ^32^, ^33^ ^]^ As shown in **Figure** **3**A,B, overexpression of NSD3 suppressed the phosphorylation of STAT3 on 727 serine site, while knockout of NSD3 facilitated STAT3 activation. However, no change had been observed in the expressions of other proteins. To further verify whether the accumulation of HK2 is regulated by STAT3 activation, we treated PC9 cells with a STAT3 inhibitor, Stattic (5 µM), for 24h, and the levels of HK2 were repressed by Stattic (Figure 3C), indicating that the expression changes of HK2 caused by NSD3 is dependent on STAT3 activation. Furthermore, we rescued the expression of NSD3 in the NSD3‐knockout PC9 cells, and the expression of total STAT3, p‐STAT3, and HK2 was examined by Western blot. As indicated in Figure 3D, NSD3‐depletion significantly promoted the expression of HK2 and the activation of p‐STAT3, whereas this effect was blunted in PC9 cells which was rescued by NSD3, verifying that NSD3 regulates the expressions of p‐STAT3 and HK2. Therefore, we addressed our hypothesis that NSD3 down‐regulated the expression of HK2 through the inhibition of the activation of STAT3. The results of the migration and invasion assay proved that knockout of NSD3 promotes the migration and invasion ability in LUAD cells, whereas the treatment of Stattic can reverse this change (Figure 3E,F). We also confirmed that the ability of colony formation and proliferation in lung adenocarcinoma cells are upregulated after NSD3 has been knockout, which can be furtherly inhibited by Stattic (Figure 3G–I). Meanwhile, we examined the glucose consumption and lactate production of PC9 cells, which suggested that the treatment of Stattic can restrain the upregulation of glucose consumption and lactate production caused by NSD3 knockout (Figure 3J,K). The expression correlation between STAT3 and HK2 was analyzed, which proved that HK2 is positively correlated with STAT3 (Figure 3L). Taken together, we concluded that NSD3 regulates HK2 through affecting STAT3 activation.

### 2.4 NSD3 Inhibited STAT3 Phosphorylation Through Binding with PPP1CB in LUAD

**Figure 4:**
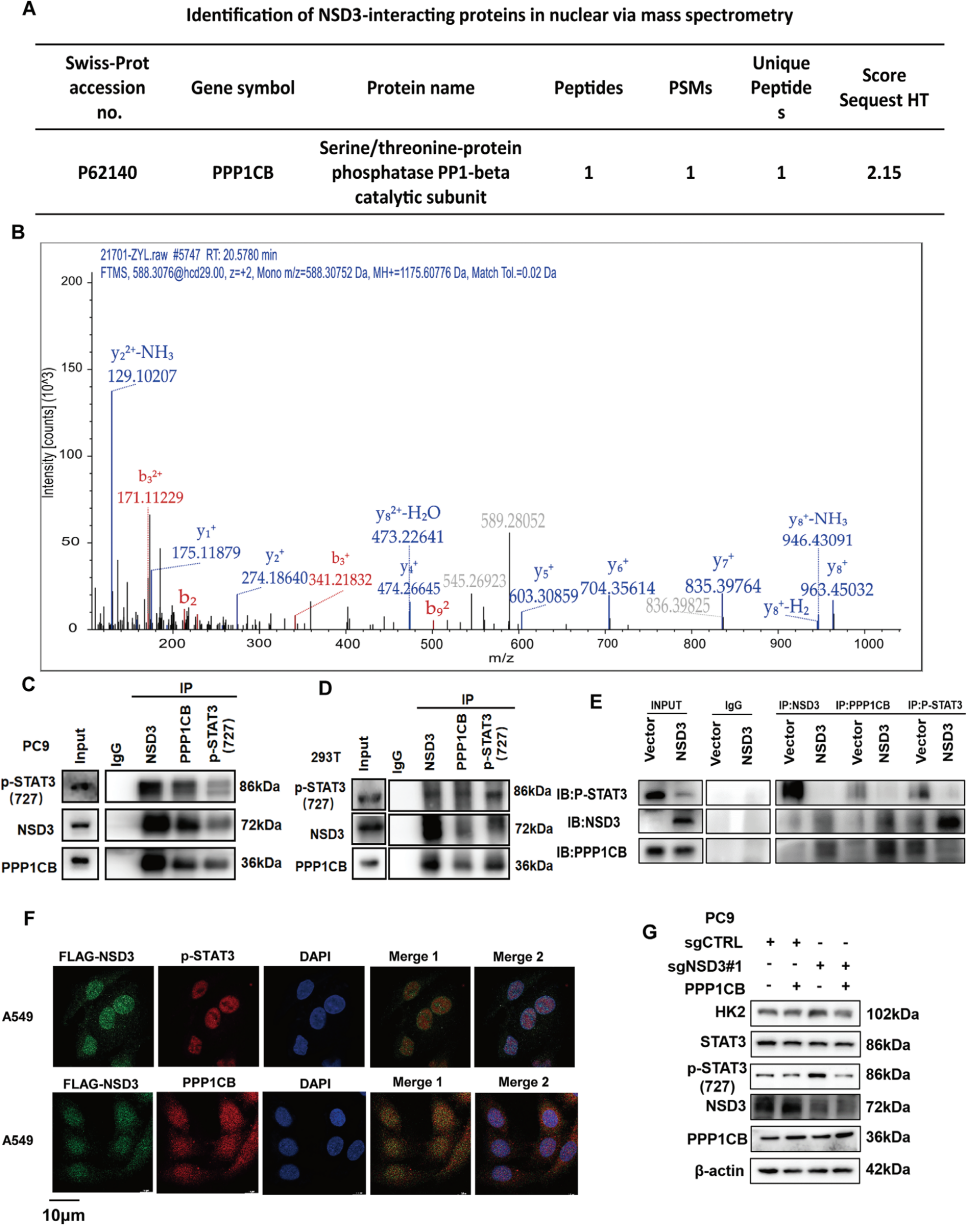
NSD3 inhibits STAT3 phosphorylation through binding with PPP1CB in LUAD. A) Mass spectrometry assay was performed, and the potentially interactive protein with NSD3 was listed. B) Mass spectrometric analysis showing interaction between NSD3 and PPP1CB in A549 cells. C) Immunoprecipitation assay were preformed to examine the endogenous interaction between NSD3, PPP1CB, and p‐STAT3 (727) in PC9. D) Immunoprecipitation assay were preformed to examine the exogenous interaction between NSD3, PPP1CB, and p‐STAT3 (727) in 293T. E) Immunoprecipitation assay were preformed to examine the binds between NSD3, PPP1CB, and p‐STAT3 were enhanced by NSD3 overexpression. F) The co‐localization of NSD3, p‐STAT3 were examined by Immunofluorescence. (Merge 1: NSD3 and p‐STAT3, Merge 2: NSD3, p‐STAT3 and DAPI) G) Control and NSD3‐knockout PC9 cells transfected with PPP1CB and analysis for the levels of HK2, p‐STAT3(727), NSD3, and PPP1CB.

To further explore the mechanisms underlying the regulation of NSD3 on STAT3 activation, a systematic mass spectrometry analysis using an anti‐NSD3 antibody was performed. Finally, we found that PPP1CB, a serine/threonine‐protein phosphatase,^[^ ^34^ ^]^ could interact with NSD3 (**Figure** **4**A,B). Then, we performed an immunoprecipitation assay with anti‐NSD3, anti‐PPP1CB, and anti‐P‐STAT3 antibodies to conform this interaction, indicating a robust endogenous and exogenous interaction between NSD3, PPP1CB, and p‐STAT3 (Figure 4C,D). Further, we conducted immunoprecipitation experiments in NSD3‐overexpressed A549 cells, and the results of Western blot showed that overexpression of NSD3 could promote the binding of NSD3, PPP1CB, and p‐STAT3 at the protein level, although overexpression of NSD3 reduces the protein abundance of p‐STAT3 (Figure 4E). Moreover, the co‐localization of NSD3, PPP1CB and p‐STAT3 was also verified by immunofluorescence (Figure 4F). It is worth mentioning that the protein level of PPP1CB was not affected by the overexpression of NSD3 (Figure S5A, Supporting Information), knockout of NSD3 (Figure S5B, Supporting Information) and rescue of NSD3(Figure S5C, Supporting Information), suggesting that there is no regulatory relationships between NSD3 and PPP1CB. To further validate the function of PPP1CB dephosphorylation on the STAT3 727 serine site, we overexpressed PPP1CB in NSD3‐knockout PC9 cells, and the results showed the existence of PPP1CB could inhibit the STAT3 activation caused by the depletion of NSD3 (Figure 4G). Meanwhile, NSD3 inhibited the STAT3 activation at 727 serine site, and the overexpressed PPP1CB could further suppress the activation of STAT3 (Figure S5D, Supporting Information). Together, these data indicate that PPP1CB is the phosphatase which binds with NSD3 to catalyze p‐STAT3 (Ser 727) dephosphorylation and inhibit HK2 transcription.

### 2.5 The Function of PPP1CB Enzyme Related to Carbon Dioxide Concentration and pH Value

**Figure 5:**
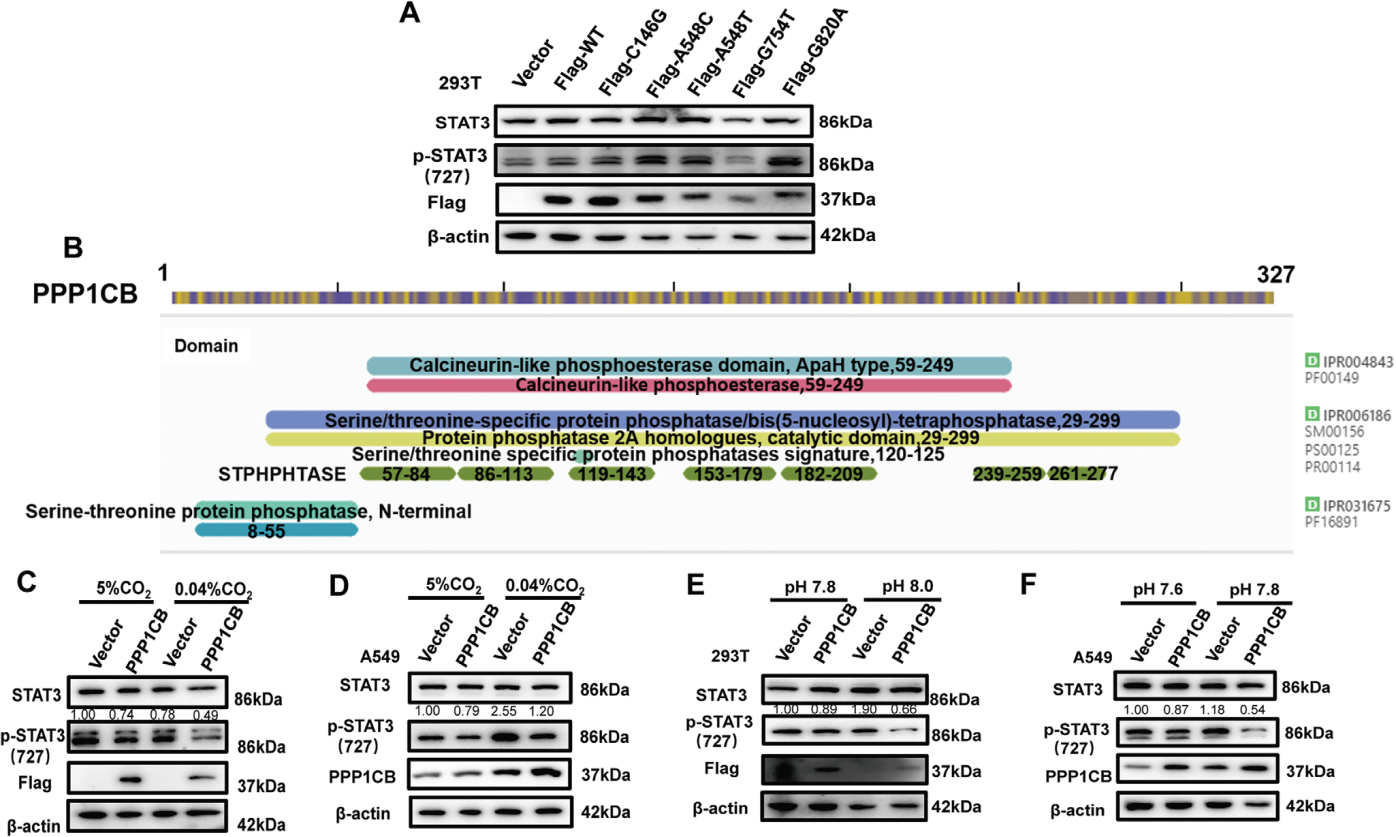
The function of PPP1CB enzyme was related to carbon dioxide concentration and pH value. A) Detection of STAT3 activation and HK2 levels in 293T cells transfected with PPP1CB‐WT, PPP1CB‐P49R, PPP1CB‐A56P, PPP1CB‐E183A, PPP1CB‐E183V, PPP1CB‐D252Y and PPP1CB‐E274K mutation plasmids. B) The predicted domain of PPP1CB by InterPro. C) Detection of STAT3 activation in A549 cells transfected with PPP1CB plasmid at different concentration of carbon dioxide. D) Detection of STAT3 activation in 293T cells transfected with PPP1CB plasmid at different concentration of carbon dioxide. E) Detection of STAT3 activation in A549 cells transfected with PPP1CB plasmid at different pH value. F) Detection of STAT3 activation in 293T cells transfected with PPP1CB plasmid at different pH value.

To find the active sites of PPP1CB protein phosphatase, we collected the mutations sites that were sequenced in disease as reported in current studies, including P49R, A56P, E183A, E183V, D252Y, and E274K, and the mutant plasmid was constructed and transfected in 293T cells. Obviously, mutation of A56P, E183A, E183V, and D252Y could inhibit the phosphatase function of PPP1CB, thus promoting the activation of STAT3 on the Ser 727 site and the transcription of HK2(**Figure** **5**A). It is worth mentioning that these mutation sites are located in the predicted phosphoesterase domain analyzed by InterPro (Figure 5B). Interestingly, recent research found that the phosphatases function of PP2C, one of the homologous family molecules of PPP1CB, is correlated with the concentration of CO_2_.^[^ ^35^ ^]^ Therefore, we speculated whether the phosphatases function of PPP1CB in LUAD is also related to CO_2_ concentration. So, we transfected the plasmid of PPP1CB into A549 and 293T cells, which proved that the function of PPP1CB is promoted by a low concentration of CO_2_ (Figure 5C,D). Furthermore, we examined the correlation between the function of PPP1CB and the pH of the culture medium. The results of Western blots showed that alkaline culture medium facilitated the function of PPP1CB (Figure 5E,F). To be specific, the phosphatases function of PPP1CB and the activation of STAT3 were promoted by low concentrations of CO_2_ and alkaline culture medium in LUAD cells. The mutation of A56P, E183A, E183V, and D252Y in PPP1CB could suppress the function of PPP1CB, which is correlated with the concentration of CO_2_ and pH value in the environment.

### 2.6 The Depletion of NSD3 Promoted the Ability of Proliferation, Migration, and Invasion in LUAD

**Figure 6:**
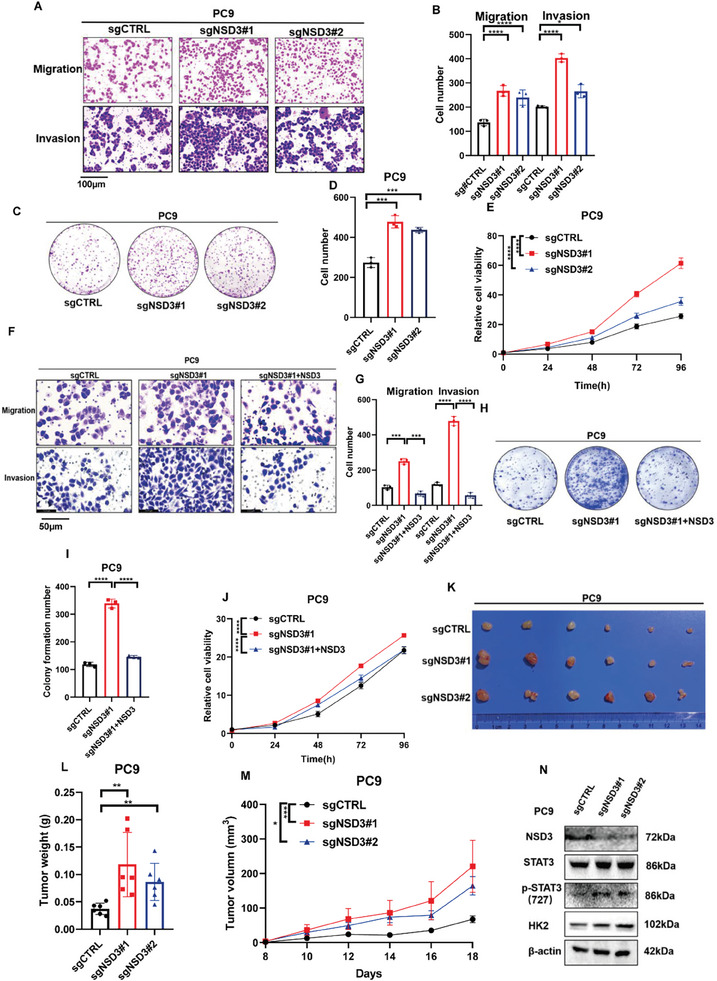
NSD3 promotes cell proliferation, migration, and invasion in PC9 cells. A) Migration and invasion experiments were performed to detect the migration and invasion ability in control or NSD3‐knockout PC9 cells. B. Qualification of (A) (n = 3 each). C) colony formation assay was performed to examine the proliferation ability in control or NSD3‐knockout PC9 cells. D) Qualification of (C) (n = 3 each). E. CCK8 assay was performed to examine the proliferation ability in control or NSD3‐knockout PC9 cells. Data are represented as mean ± S.E.M. (n = 5). P values are obtained by two‐way ANOVA. F) After NSD3 was transfected, migration and invasion experiments were performed to detect the migration and invasion ability in control or NSD3‐knockout PC9 cells. G) Qualification of (F) (n = 3 each). H) After NSD3 was transfected, colony formation experiments were performed to detect the proliferation ability in control or NSD3‐knockout PC9 cells. I) Qualification of (H) (n = 3 each). J) After NSD3 was transfected, CCK8 experiments were performed to detect the proliferation ability in control or NSD3‐knockout PC9 cells. Data are represented as mean ± S.E.M. (n = 5). P values are obtained by two‐way ANOVA. The xenograft tumor formation assay was performed to determine the tumor formation ability in in control or NSD3‐knockout PC9 cells (Figure 5 K, L, and M). N) Western blot was performed to test NSD3,p‐STAT3, and HK2 expression in xenograft from NSD3‐knockout PC9 cells or control cells. scale bar, 200 µM. ^*^ *p * < 0.05; ^**^ *p* < 0.01; ^***^ *p* < 0.001; and ^****^ *p* <  0.0001.

We next validated the role of NSD3 in vitro. The migration and invasion ability was enabled after NSD3 was knockout (**Figure** **6**A,B). Moreover, NSD3 knockout significantly promoted colony formation ability(Figure 6C,D) and facilitated cell growth rate (Figure 6E). Furthermore, the rescue of NSD3 could suppress the progression in the ability of migration, invasion (Figure 6F,G), colony formation (Figure 6H,I), and proliferation (Figure 6J) caused by NSD3‐knockout. A xenograft mouse model was established in nude mice to explore whether NSD3 also plays a role in LUAD progression in vivo. More extensive tumors were formed in nude mice injected with NSD3‐knockout PC9 cells compared to tumors in nude mice injected with control PC9 cells (Figure 6K,L). The tumor from the mouse injected with NSD3‐knockout PC9 cells grew faster and more extensive than the tumor of control PC9 cells (Figure 6M). The results of Western blot and IHC demonstrated that the expressions of p‐STAT3 and HK2 were promoted in tumor from nude mice with NSD3‐knockout PC9 cells (Figure S6, Supporting Information). Meanwhile, the levels of Ki‐67 in tumor from nude mice with NSD3‐knockout PC9 cells were higher than in the control group, suggesting that the low levels of NSD3 are positively correlated with a stronger proliferation ability (Figure S6, Supporting Information). Altogether, these results proved that NSD3 plays a vital role in proliferation, migration, and invasion as a potential tumor suppressor in LUAD. Taken together, our data showed that knockout of NSD3 promoted tumor growth of LUAD in vivo and in vitro.

### 2.7 NSD3 Impeded Cell Proliferation, Migration, and Invasion in LUAD

**Figure 7:**
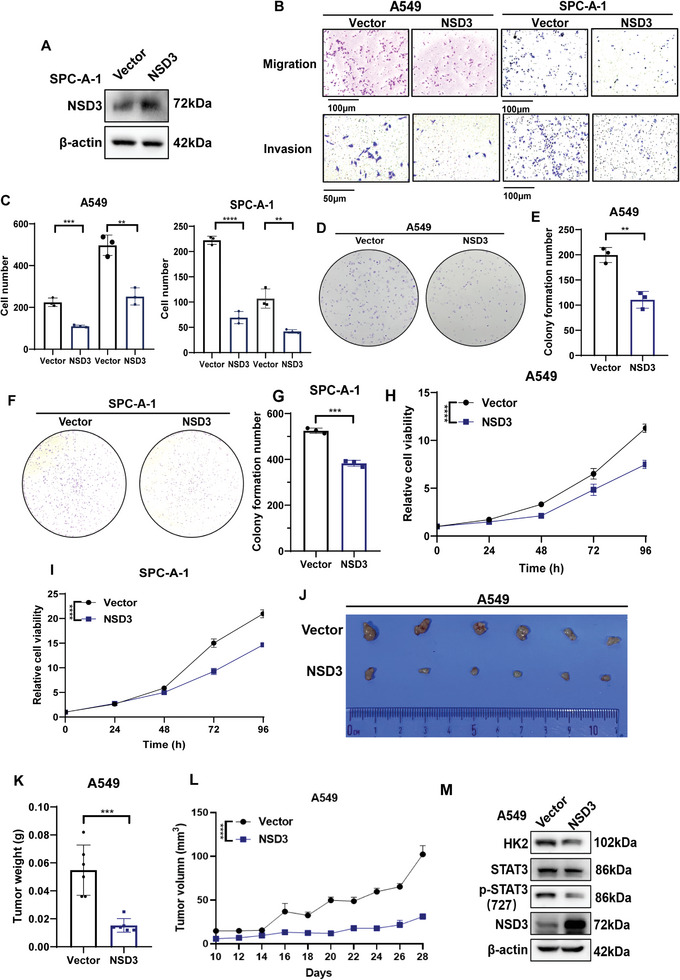
NSD3 suppressed cell proliferation, migration, and invasion in A549 and SPC‐A‐1 cells. A) Western blot was performed to detect the levels of NSD3 in vector and NSD3‐overexpressed SPC‐A‐1 cells. B) Migration and invasion experiments were performed to detect the migration and invasion ability in vector or NSD3‐overexpressed A549 and SPC‐A‐1 cells. C) Qualification of (B) (n = 3 each) D. Colony formation assay was performed to examine the proliferation ability in vector or NSD3‐overexpressed A549 cells. E. Qualification of (D) (n = 3 each). F) Colony formation assay was performed to examine the proliferation ability in vector or NSD3‐overexpressed SPC‐A‐1 cells. G. Qualification of (F) (n = 3 each). H) CCK8 assays were performed to test the proliferation ability in vector or NSD3‐overexpressed A549 cells. Data are represented as mean ± S.E.M. (n = 5). P values are obtained by two‐way ANOVA. I. CCK8 assays were performed to test the proliferation ability in vector or NSD3‐overexpressed SPC‐A‐1 cells. Data are represented as mean ± S.E.M. (n = 5). P values are obtained by two‐way ANOVA. The xenograft tumor formation assay was performed to determine the tumor formation ability in vector or NSD3‐overexpressed PC9 cells (J, K, and L). M. Western blot was performed to test NSD3, p‐STAT3, and HK2 expression in xenograft from NSD3‐knockout PC9 cells or control cells. ^*^ *p *< 0.05; ^**^ *p* < 0.01; ^***^ *p* < 0.001; and ^****^ *p* < 0.0001.

To comprehensively clarify the functions of NSD3 in LUAD, overexpressed NSD3 cell lines were established in A549 and SPC‐A‐1 cells (**Figure** **7**A; Figure S4C, Supporting Information). The migration and invasion abilities of NSD3‐overexpressed A549 and SPC‐A‐1 cells were suppressed compared to control cells (Figure 7B,7C). At the same time, the consequences of colony formation experiments (Figure 7D,7G) and CCK8 assays (Figure 7H,I) pointed out that the high expression of NSD3 inhibits the proliferation of LUAD cells. The overexpressed of NSD3 significantly suppressed tumor growth (Figure 7J‐L), indicating that NSD3 reduces tumorigenesis in vivo. The levels of HK2 and p‐STAT3 (727) were inhibited when NSD3 was overexpressed in tumor from nude mice examined by Western blot and IHC (Figure 7M; Figure S6, Supporting Information). Together, these results suggested that NSD3 functions as a tumor suppressor in LUAD.

## 3 Discussion and Conclusion

**Figure 8:**
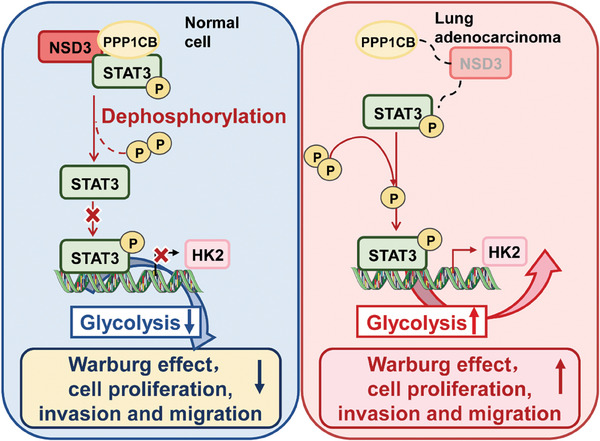
Research pattern diagram of histone methyltransferase NSD3 regulating glycolysis and lung cancer progression in lung adenocarcinoma. Under normal circumstances, the high expression of NSD3 can bind to PPP1CB, dephosphorylate phosphorylated STAT3, and inhibit the activated STAT3 from entering the nucleus and binding to the transcription initiation region of HK2, thereby inhibiting the transcription of HK2, leading to the decrease of glycolysis level, proliferation, migration and invasion ability of cells. In lung adenocarcinoma cells, the expression of NSD3 is decreased or its activity is decreased due to the mutation of NSD3, and its binding with PPP1CB also reduces the dephosphorylation effect of phosphorylated STAT3. The accumulated phosphorylated STAT3 can bind to the transcription start region of HK2 in the nucleus, resulting in the transcription of HK2. Furthermore, it can promote cell glycolysis and improve the proliferation, migration, and invasion ability of lung adenocarcinoma cells.

This research systematically clarified the regulation roles of NSD3 in lung adenocarcinoma progression and metabolization. Systematic analyses of clinical samples and functional experiments proved that high levels of NSD3 associated with poor prognosis and tumor progression mediated by STAT3 activation. As a meaningful histone methyltransferase, our research demonstrated that NSD3 forms a protein trimer with PPP1CB and p‐STAT3, promotes the conjuction between PPP1CB and p‐STAT3, and facilitates the dephosphorylation of p‐STAT3. The formation of this trimer will further cause the inhibition of HK2 transcription and glycolysis process (Figure 8). Meanwhile, we proved that the dephosphorylation function of PPP1CB is related to the the concentration of CO_2_ and pH value in the culture environment, and the mutations of A56P, E183A, and D252Y in PPP1CB resulted in the inactived enzyme functions. Consequently, NSD3 serves as a non‐epigenetic regulator in lung adenocarcinoma development, indicating that NSD3 might be a therapeutic target in lung adenocarcinoma treatment.

Our research comprehensively clarified that NSD3 significantly inhibited the malignant behavior of lung adenocarcinoma cells. Nowadays, growing research shows that NSD3 is extremely important for tumorigenesis. It has been proved that methylation of H3K36 induced by NSD3 promotes breast cancer initiation and metastasis via NOTCH signaling.^[^ ^18^, ^36^ ^]^ Meanwhile, the upregulation of NSD3 was positively correlated with the proliferation and migration ability in colorectal and pancreatic cancer.^[^ ^37^, ^38^ ^]^ However, there are limited reports on the mechanism of NSD3 in the development of LUAD. In our study, we found NSD3 was lowly expressed in LUAD, and low levels of NSD3 were correlated with higher tumor grade and poor survival, suggesting that low levels of NSD3 prompt a poor prognosis. Interestingly, our study reconfirmed the expression difference of NSD3 between LUAD and LUSC: NSD3 was significantly upregulated in LUSC but downregulated in LUAD. In order to clarify the underlying mechanism of this difference in expression, we performed mutation analysis (including amplification, deletion, nonsense mutation, etc.) on TCGA LUSC and LUAD databases. The results suggested that the difference in the expression of NSD3 between LUSC and LUAD may be related to the mutation frequency and mutation status of the chromosome region 8p11.2 of NSD3 in the two diseases and the gene mutation background of LUSC and LUAD, respectively.(**Figure** **8**)

Moreover, our research elucidated a new model in which NSD3 is involved in STAT3 activation and the transcription of glycolysis enzymes as a non‐epigenetic regulator. It is noteworthy that previous studies have mainly focused on how the epigenetic regulatory role of NSD3 regulates squamous cell lung cancer and breast cancer,^[^ ^6^, ^18^ ^]^ raising the need to understand the comprehensive impact of NSD3 on tumor initiation and progression. On the basis of our findings, it could be proposed the non‐epigenetic function of NSD3 which further revealed the correlation between the non‐epigenetic function of NSD3 and tumor cell metabolism.

Since NSD3 has no known protein kinase or phosphatase function, we reasoned that NSD3 might regulate STAT3 phosphorylation by binding to a protein kinase or phosphatase. Our research found that dephosphorylation of p‐STAT3 facilitated by PPP1CB could further inhibit the transcription of HK2, suggesting a new model that the dephosphorylation function of PPP1CB affects STAT3 activation and its enzyme activation mutation sites (A56P, E183A, E183V, and D252Y). It has been reported that PPP1CB facilitated the adipogenesis in 3T3‐L1, which could be prevented by Chebulinic Acid.^[^ ^39^, ^40^ ^]^ The mutation of PPP1CB and fusion of PPP1CB‐ALK was considered significant risks for progression in glioma, chronic lymphocytic leukemia, and hepatocellular carcinoma.^[^ ^41^, ^42^, ^43^ ^]^ The roles of PPP1CB in lung adenocarcinoma remain unknown. We innovatively demonstrated that NSD3 can form a protein trimer by combining with PPP1CB and p‐STAT3, which is promoted by the overexpression of NSD3, eventually promote the phosphorylation process of p‐STAT3. However, we noticed the expression of PPP1CB was not affected by the overexpression of NSD3, which suggests that there is no clear regulatory relationship between NSD3 and PPP1CB and NSD3 play its role in STAT3 actvation via the forming of NSD3/PPP1CB/p‐STAT3 dimer in lung adenocarcinoma.

Many previous studies have shown that the function of phosphatase is related to the concentration of carbon dioxide. Environmental microbiology studies have shown that high CO_2_ concentration facilitates phosphatase activity in soil enzymes.^[^ ^44^ ^]^ In detail, the structure of Receptor Protein Tyrosine Phosphatase γ can be affected by CO_2_/ HCO_3_^−^ concentrations.^[^ ^45^ ^]^ Moreover, H_2_O_2_ mediates the dysfunction of Protein Tyrosine Phosphatase, while elevated CO_2_/HCO_3_^−^ enhances this pathophysiological process.^[^ ^46^ ^]^ IDRs in PP2C featuring polar amino acids of serine/threonine are affected by the concentration of CO_2,_ not HCO_3_^−^, and thus the enzyme activities are altered.^[^ ^35^ ^]^ We demonstrated that the activation of STAT3 was influenced by the concentration of CO_2_, and the dephosphorylations of PPP1CB were amplified. Since the concentration of CO_2_ in the environment affects the pH of the culture medium environment, we further test the relationship between pH value and function of PPP1CB. Similar results to CO_2_ concentration were obtained in different pH values, which suggested that the phosphorylation function of PPP1CB and the activation of STAT3 were upregulated in an alkaline environment. This phenomenon indicated that the regulation of CO_2_ concentration and pH value on STAT3 activation may have different mechanisms in different diseases, which deserves a deeper exploration.

Our study established a new molecular mechanism underlying the transcription of HK2 via the formation of NSD3/PPP1CB/p‐STAT3 complex. Since NSD3 and p‐STAT3 were located in the nucleus, we suggested that the construction of this complex facilitated PPP1CB enter the nucleus, further promoted the combination of PPP1CB and p‐STAT3, ultimately facilites the dephosphorylation p‐STAT3. As an important transcription regulator, STAT3 signaling pathway also plays a crucial role in tumor immunity, inflammation, and antophagy.^[^ ^47^, ^48^, ^49^, ^50^ ^]^ The phosphorelated state of STAT3 was correlated with the transcription of downstream genes, which could be affected by IL‐6 and other regulators, suggesting an intermolecular regulatory network.^[^ ^51^, ^52^ ^]^ Meanwhile, the STAT3 signaling pathway could affect HK2 transcription and further affect glycolysis, which could be regulated by circCUL3.^[^ ^29^ ^]^ However, whether NSD3 can regulate the STAT3/HK2 pathway has not been reported. In this project, we innovatively found and discussed that NSD3 can inhibit STAT3 activation in lung adenocarcinoma and then inhibit HK2 gene expression and glycolysis process by the formation of NSD3/PPP1CB/p‐STAT3 complex.

Accumulating studies have focused on the benefits of NSD3 targets for targeted therapy. It has been proved that a PROTAC‐targeted NSD3, MS9715, can prevent the progression of tumor of NSD3‐dependent.^[^ ^53^ ^]^ BI‐9321 has also been proven to be the antagonist of the NSD3‐PWWP1 domain.^[^ ^54^ ^]^ However, agonists targeting NSD3 have not yet been developed, which impedes the benefits of NSD3 targeting the targeted therapy of lung adenocarcinoma. Therefore, further investigation is needed for the benefit of lung adenocarcinoma patients with low NSD3 expression.

Together, our research clarifies the crucial role of NSD3 in lung adenocarcinoma that is correlated with NSD3/PPP1CB/p‐STAT3 dimer formation, revealing the correlation between non‐epigenetic functions of NSD3 and tumor cell metabolism, suggesting the potential of NSD3 in targeted therapy in lung adenocarcinoma.

## 4 Experimental Section

### Cell Culture and Reagents

Normal lung cell lines, BEAS‐2B, were purchased from the ATCC. The lung cancer cell lines H358 (ATCC: CRL‐5807™) and A549 (ATCC: CCL‐185™) were purchased from the ATCC. The human embryonic kidney epithelial cell line 293T and the lung cancer cell lines PC9, H23, H520, H1299, SPC‐A‐1, and 95C were obtained from the Cancer Research Institute of Central South University. 293T cells were cultured in DMEM (Gibco), and others were cultured in RPMI 1640 (Gibco). All media were added with 10% fetal bovine serum (FBS), and all cells were cultured in an atmosphere of 5% CO_2_ at 37 °C. The results for mycoplasma contamination in all cell lines were negative. Stattic (Sellck, s7024) was used to inhibit the activation of STAT3.

### Plasmids, sgRNA and siRNA

NSD3 overexpressing plasmids were constructed by MSCV‐NSD3‐short (Addgene, #72552) into the pLVX‐EF1α‐IRES‐Puro vector (cat#631988, Clontech). PPP1CB overexpressing plasmids (RC201142) were purchased from Origenes. Lentiguipuro sgRNA clones targeting human NSD3 as follows: #1 (F: CACCGCCGCACTTG TGTGCCGCCTC, R: AAACGAGGCGGCACACAAGTGCGGC), #2 (F: CACCGTGTCACAGTTGGGAGAGTAA, R: AAACTTACTCTCCCAACTGTG ACAC). For HK2 RNA interference, the human HK2‐targeting siRNA sequence as follow: #1(a: AGAUACUGGUCAACCUUCUGC s:GCAGAAGGUUGACCAGU AUCU). The control constructs lentiGuide‐Puro plasmid (puromycin resistance) were purchased from the Addgene. All the lentiviral particles were produced in 293T cells. SPC‐A‐1, PC9 and A549 cell lines were selected by puromycin at a concentration of 1 µg mL^−1^.

### Western Blot and Immunoblotting Antibodies

Cells samples were lysed by IP buffer. 20 µg proteins were electrophoresis and transferred into polyvinylidene fluoride (PVDF) transmembrane. The following primary antibodies were used to detect the proteins on NC membranes overnight at 4 °C: anti‐NSD3 (proteintech, 11345‐1‐AP, 1:1000), Anti‐β‐actin (sigma, A5441, 1:10000), Anti‐HK2 (proteintech, 22029‐1‐AP, 1:2000), Anti‐STAT3 (ABclonal, A19566, 1:1000), Anti‐Phospho‐STAT3‐S727 (ABclonal, AP0715, 1:1000), Anti‐p53 (Santa cruz, sc‐126, 1:1000),Anti‐HIF‐1α (Cell signaling technology, #48085, 1:1000), Anti‐C‐MYC (Cell signaling technology, #18583, 1:1000), Anti‐PPP1CB (proteintech,10140‐4‐AP, 1:1000). Horseradish peroxidase (HRP)‐conjugated secondary antibody was used to detect the target protein.

### IHC

Lung adenocarcinoma tissue samples were obtained from the Department of Pathology of Xiangya Hospital. IHC analysis of paraffin‐embedded tissue samples obtained from patients with lung cancer was performed as described previously.^[^ ^55^ ^]^

### Real‐Time Quantitative PCR (RT‐qPCR)

Total RNAs were extracted by RNAeasy™ Plus Animal RNA Isolation Kit with Spin Column (Beyotime, R0032), and 1 µg total RNA was reversed to cDNA by gDNA Erase and PrimeScript RT Reagent Kits (TAKARA Biotechnology, Dalian, China). Hieff qPCR SYBR Green Master Mix (YEASEN) was used to detect the expression status of candidate genes and β‐actin. The primer we used were listed below:ALDOA(F: GACACTCTACCAGAAGGCGGAT and R: GACACTCTAC CAGAAGGCGGAT), ENO1(Fe: AGTCAACCAGATTGGCTCCGTG and R: CACAACCAGGTCAGCGAT GAAG), GLUT1(F: TTGCA GGCTTCTCCAACTGGAC and R: CAGAACCAGGA GCACAGTGAAG), HK2(F: GAGTTTGACCTGGATGTGGTTGC and R: CCTCC ATGTAGCAGGCATTGCT), LDHA(F: GGATCTCCAACATGGCAGCCTT and R: AGACGGCTTTCTCCCTCTTGCT), PKM2(F: ATGGC TGACACATTCCTGGAGC and R: CCTTCAACGTCTCCACTGATCG), PFKFB3(F: GGCAGGAGAA TGTGCTGGTCAT and R: CATA AGCGACAGGCGTCAGTTTC), NSD3(F: CAG ACGTTTCTGATG TGCAGTCC and R: CTCCAGGTGAAAGTGTTTGCAGC).

### Co‐Immunoprecipitation Assay

Cell samples were lysed in IP buffer for 1 h, and 1000 µg total proteins were prepared for follow‐up experiments. 1 µg primary antibody was added to sample and 1 µg corresponding IgG was added for negative control. 50 µg total proteins were prepared for INPUT. The 1000 µg total proteins and 1 µg primary antibody or 1 µg corresponding IgG were mixed and rotated at 4 °C for 1 h, and 20 µL Protein A + G Agarose was added into the tubes and rotated at 4 °C overnight. The beads were washed with cold PBS for 3500rpm 5 min for three times. Finally, gently discard the supernatant and add 40 µL 1X loading.

### Glucose Consumption and Lactate Production

Cells (5 × 10^5^) were plated in a 6‐well plate and cultured in 3 mL RPMI‐1640 medium with 10% FBS. After 48 h, 1 mL cell culture supernatant was collected and centrifuged at 800 rpm for 5 mins. The supernatant was collected and sent to the department of clinical laboratory in Xiangya hospital to measure glucose consumption and lactate production.

### Migration and Invasion Assay

In the migration assay, 2 × 10^4^ cells in 200 µL medium with 1% FBS were planted in inserts placed in 24‐well plates, and 800 µL medium with 10% FBS were added to 24‐well plates. After 24h incubation, fixed the bottom of the wells with 4% paraformaldehyde and stained it with crystal violet. Three fields were counted for each well in the microscope.

In the invasion assay, 180 µL medium and 20 µL Matrigel basement membrane matrix was mixed (medium: Matrigel = 9:1) and the 200 µL mixture was placed in the transwell inserts for 4h before cells were added at 37 °C. 6 × 10^4^ cells in 200 µL medium with 1% FBS were planted in inserts placed in 24‐well plates, and 800 µL medium with 10% FBS were added to 24‐well plates. After 48h incubation, fixed the bottom of the wells with 4% paraformaldehyde and stained it with crystal violet. Three fields were counted for each well in the microscope.

### Colony Formation Assay

1000 cells were planted in a 6‐wells plate, and 3 mL mediums with 10% FBS were added. Mediums were replaced every 3 days. After incubation of 10 days, the dishes were washed with PBS, fixed with 4% paraformaldehyde, and stained with crystal violet. Colonies' numbers were counted by ImageJ.

### Cell Counting Kit‐8 (CCK‐8) Assay

Eight hundred cells were planted in a 96‐wells plate, and 100µL mediums with 10% FBS were added. 10 µL Cell counting kit‐8 (bimake, B34302) was added and mixed at 0h, 24h, 48h, 72h, and 96h. The 450nm OD was measured after 1h incubation.

### Point Mutation

Collect the mutation site and condition from uniport (https://www.uniprot.org/uniprot/P62140). Mutation primers were obtained from PrimerX. Mutation plasmids were obtained from PCR procedure with corresponding primers. The monoclonal colonies were obtained by bacterial transformation. Finally, sequencing verified the successful construction of mutant plasmids.

### Cell Culture Under Different CO_2_ Conditions

5×10^5^ cells were planted in two different 6‐well plate. After 24 h cultivation at 37 °C 5% CO_2_, one plate was incubated at 37 °C 0.04% CO_2_ for another 3 h. Another plate was incubated at the original condition for the same 3 h.

### Immunofluorescence

A549 cell were plated on the cover slips in 6‐well plate for 48 h, and then fixed with 4% paraformaldehyde, permeabilized with 0.5% Triton X‐100, and blocked with 5% goat serum. After overnight incubation with primary antibodies at 4  °C, cells were incubated with corresponding secondary antibody at 37  °C for 2  h in dark. With counterstaing with DAPI, cells were observed and photos were obtained by Laser scanning confocal microscope (Leica, SP8‐DMIL).

### Xenograft Tumor

The xenograft tumor formation assay was performed essentially as previously described.^[^ ^56^, ^57^, ^58^, ^59^ ^]^ All procedures for the animal study were approved by the Institutional Animal Care and Use Committee of the Central South University of Xiangya School of Medicine and conformed to the legal mandates and federal guidelines for the care and maintenance of laboratory animals. Five‐week‐old male BALB/c athymic mice were purchased from the Hunan SJA Laboratory Animal Co. Ltd. and housed in dedicated pathogen‐free barrier facilities. Mice were injected with 5×10^6^ cells in the subcutaneous tissue. The weight of the mice was recorded every 3 days. The tumor volumes were calculated every 3 days using the following equation: tumor volume (mm^3^) = π/6 × (tumor length) × (tumor width)^2^. Data were analyzed using Student's t‐test; a *p*‐value < 0.05 was considered significant.

### Study Approval

The research was authorized by Central South University Xiangya hospital's ethics committe (2023030109). Lung adenocarcinoma patient samples was obtained from the Xiangya Pathologic Anatomy Service's files. The use of animal models in this work was approved by Central South University's institutional Animal Care and Use Committee. All of the participating medical facilities’ institutional review boards gave their approval to the study. Before enrollment, all study participants signed a written informed consent form.

### Reproducibility and Statistics

All experiments, except for experiments involving in nude mice, were repeated at least three times. The data were presented as the means ± SD of at least three independent experiments. Two‐tailed unpaired Student t‐tests were used for the intergroup comparisons. Kaplan‐Meier method was chosen to assess patients’ survival. ^*^p < 0.05; ^**^p  < 0.01; ^***^p < 0.001; and ^****^p < 0.0001.

## Conflict of Interest

The authors declare no conflict of interest.

## Supporting information

Supporting Information

## Data Availability

The data that support the findings of this study are available from the corresponding author upon reasonable request.
