# Supplementary material for: Histones Methyltransferase NSD3 Inhibits Lung Adenocarcinoma Glycolysis Through Interacting with PPP1CB to Decrease STAT3 Signaling Pathway
Source: Adv Sci (Weinh). 2024 Aug 9;11(38):2400381. doi: 10.1002/advs.202400381 (PMC11481231; doi:10.1002/advs.202400381)
Supplement: Supplementary file 1 — Supporting Information [file ADVS-11-2400381-s001.docx]

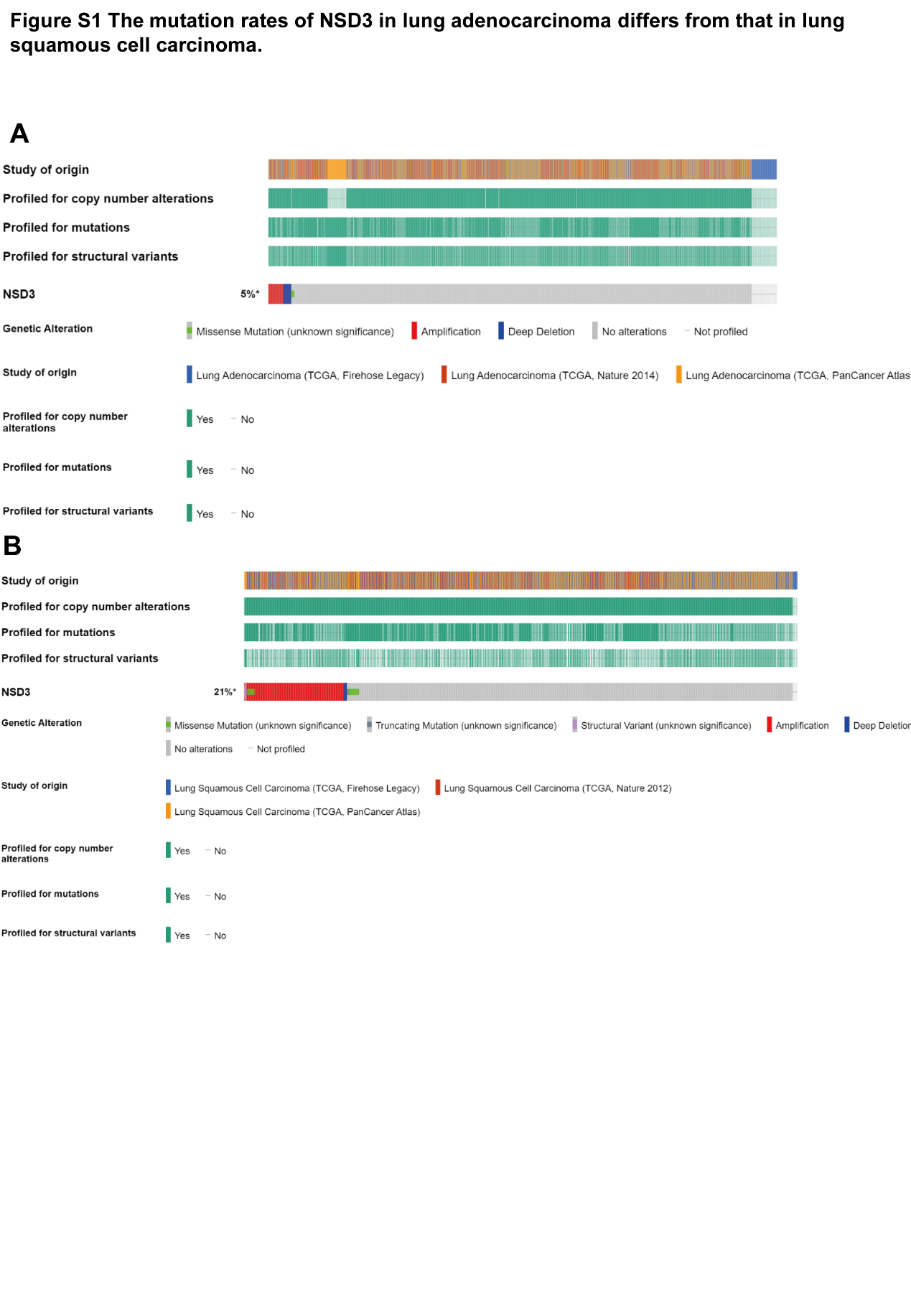


**Figure S1. The mutation rates of NSD3 in lung adenocarcinoma differs from that in lung squamous cell carcinoma.**

A, B. Analysis of NSD3 in lung cancer datasets from TCGA indicates that the mutation frequency of NSD3 in lung adenocarcinoma was significantly lower than that in lung squamous cell carcinoma.


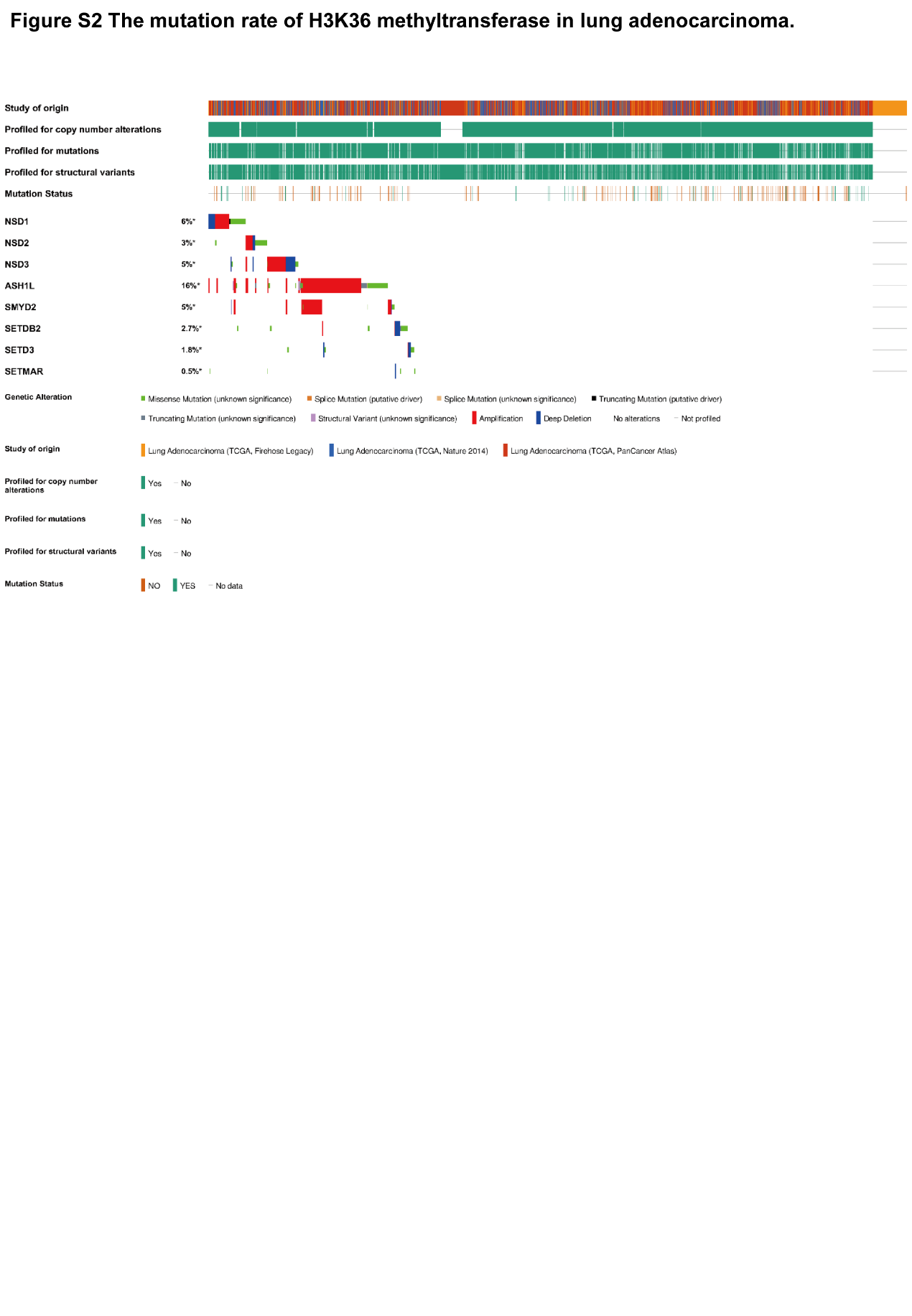


**Figure S2. The mutation rate of H3K36 methyltransferase in lung adenocarcinoma.**


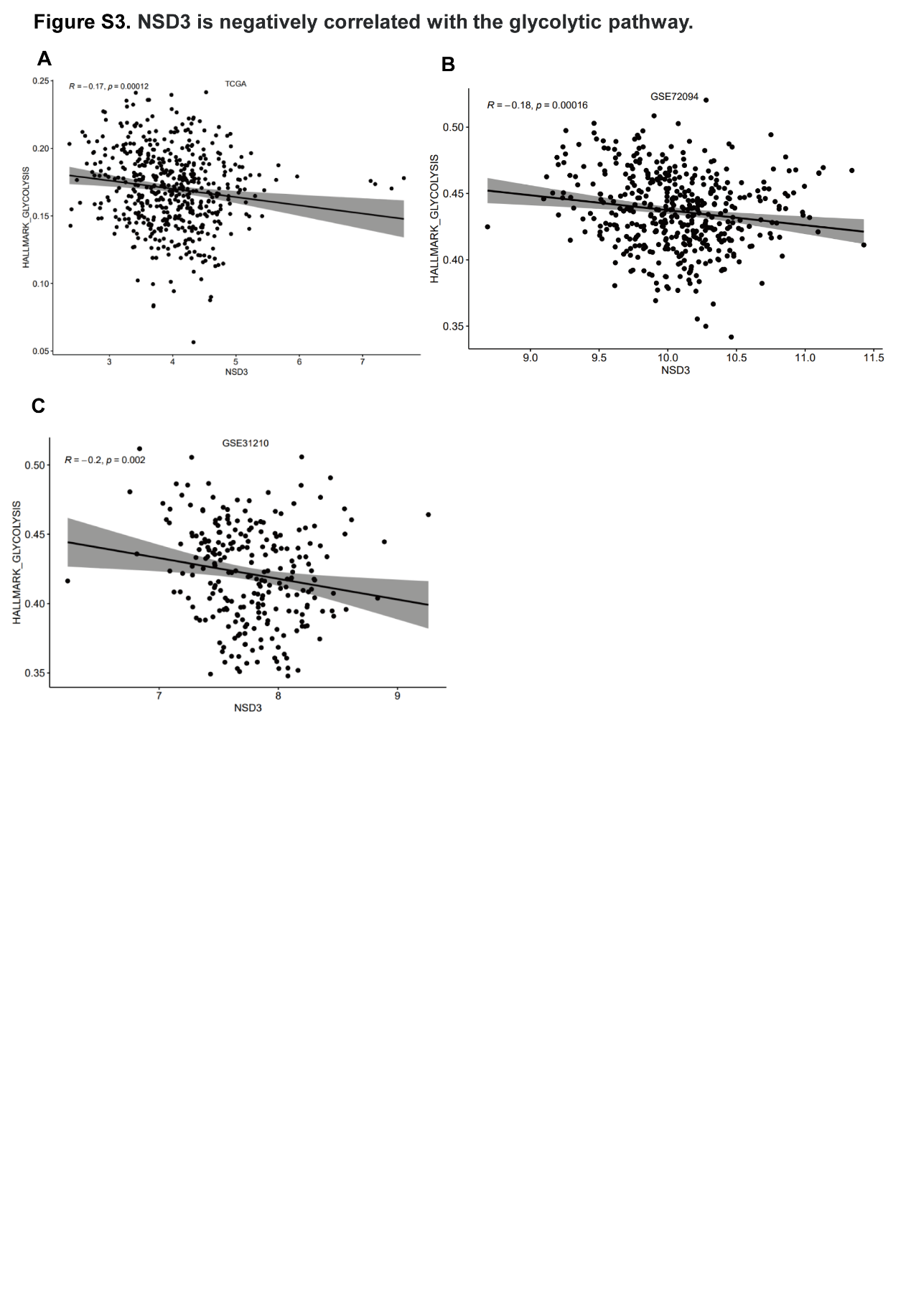

**Figure S3. The expression of PKM2 was not affected when NSD3 is overexpressed or knockout.**

A-C TCGA (P = 0.00012, R = -0.17), GSE31210 (P = 0.002, R = -0.2) and GSE72094 (P =0.00016, R = -0.18) were negatively correlated with glycolysis pathway.


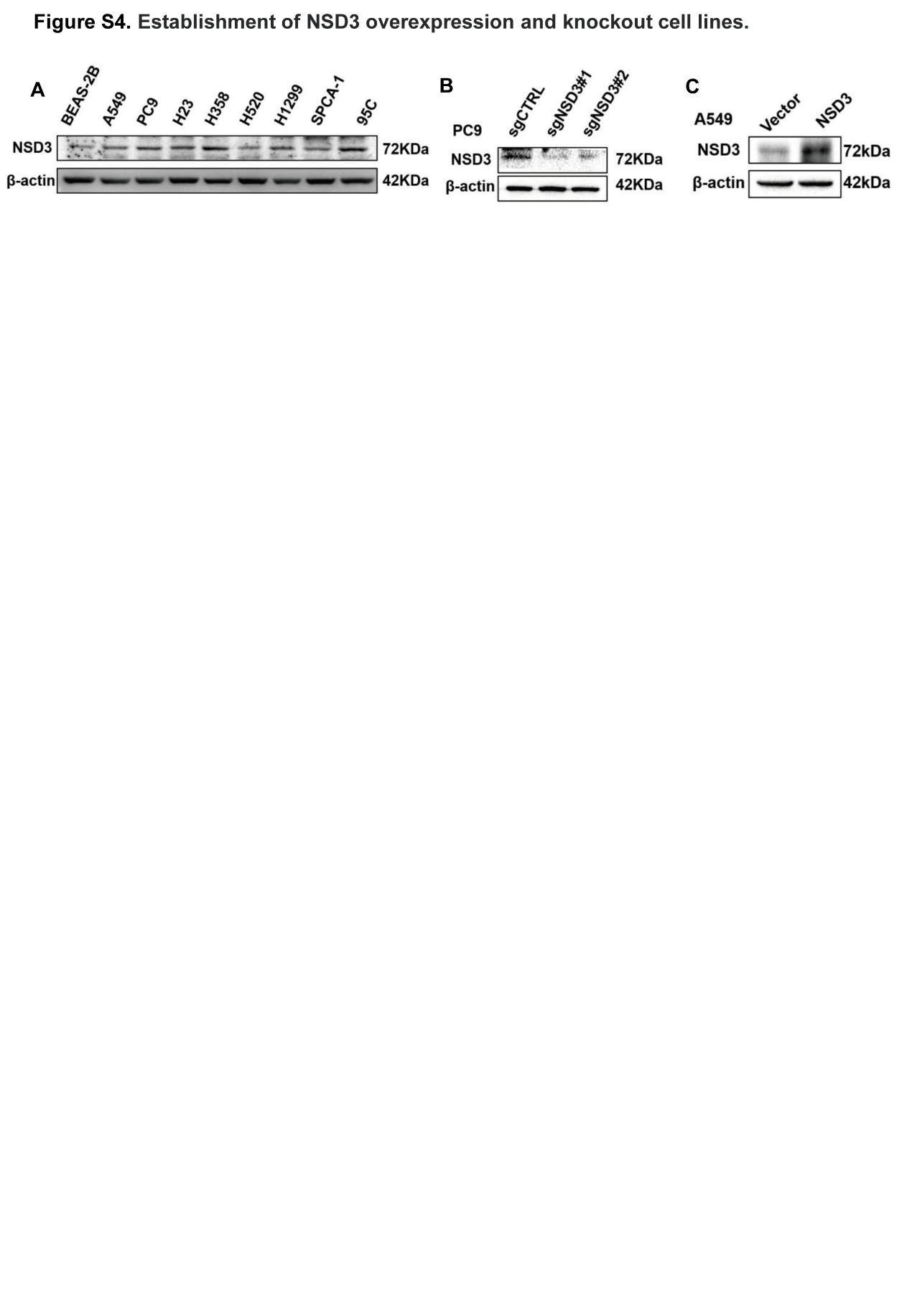


**Figure S4. Establishment of NSD3 knockout and overexpression lung adenocarcinoma cell lines.**

A. Western blot analysis was performed to detect the expression of NSD3 in lung bronchial epithelial cells and lung cancer cells. B. Western blot analysis was performed to clarify the establishment of NSD3 knockout cell line in PC9. C. Western blot analysis was performed to clarify the establishment of NSD3 overexpression cell line in A549.


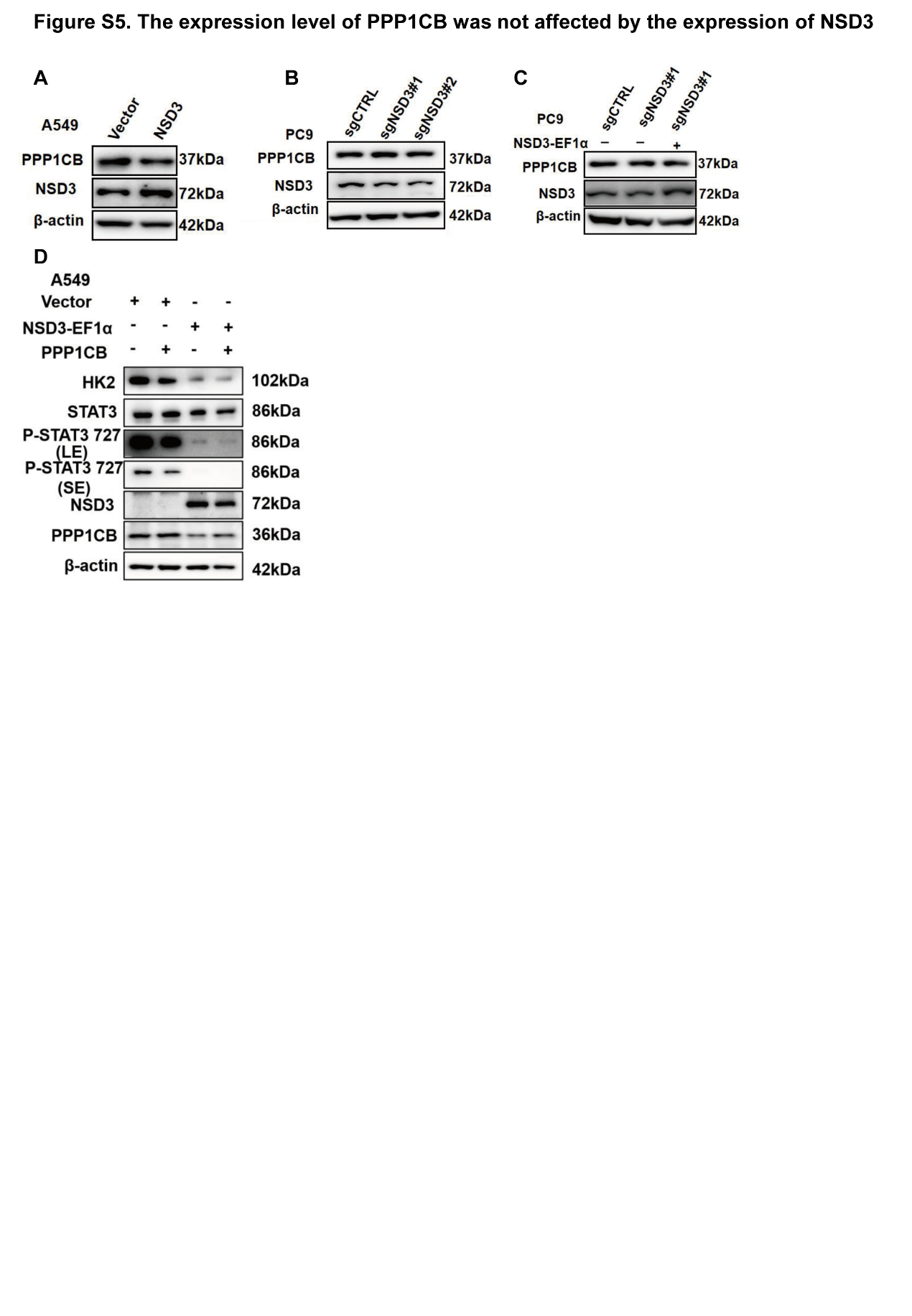


**Figure S5. The expression level of PPP1CB was not affected by the expression of NSD3**

A. Western blot analysis was performed to detect the expression of PPP1CB in vector or NSD3-overexpressed A549 cells. B. Western blot analysis was performed to detect the expression of PPP1CB in sgControl or NSD3-knockout PC9 cells. C. Western blot analysis was performed to detect the expression of PPP1CB in sgControl or NSD3-knockout PC9 cells rescued with NSD3. D. Control and NSD3-overexpressed A549 cells transfected with PPP1CB and analysis for the levels of HK2, p-STAT3(727), NSD3 and PPP1CB.


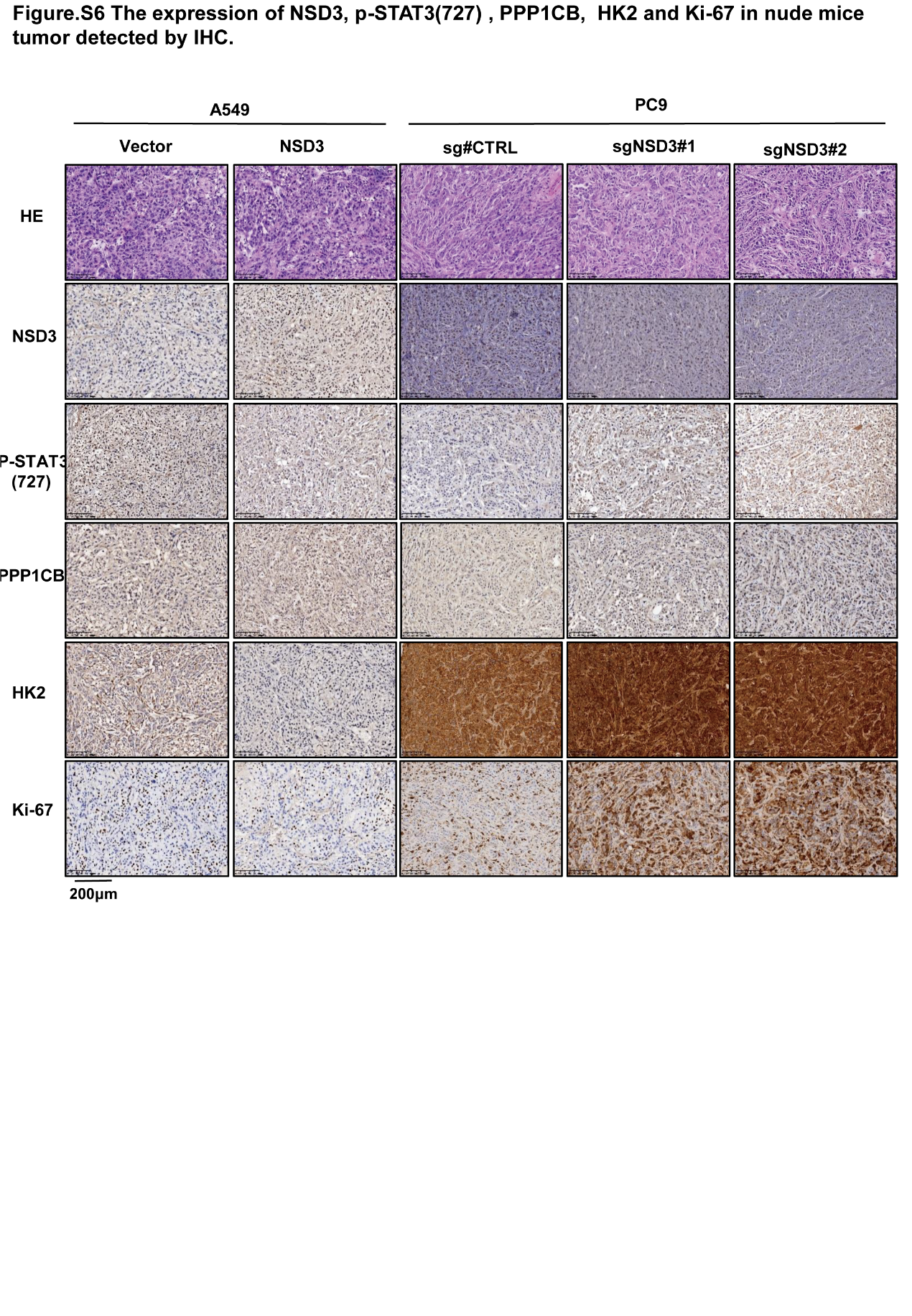


**Figure S6. The expression of NSD3, p-STAT3(727), PPP1CB, HK2 and ki-67 in nude mice tumor detected by IHC.**

Hematoxylin and Eosin staining, immunohistochemistry staining for the indicated proteins of a representative primary xenograft originating from NSD3-knockout PC9 cells or control cells and NSD3-overexpressed A549 cells or control cells. scale bar, 200 μm.
